# Challenges and limitations of using ventilator-free days as an outcome in critical care trials

**DOI:** 10.62675/2965-2774.20240088-en

**Published:** 2024-04-22

**Authors:** Alejandro Bruhn, Eduardo Kattan, Alexandre Biasi Cavalcanti

**Affiliations:** 1 Pontificia Universidad Católica de Chile Facultad de Medicina Department of Intensive Medicine Santiago Chile Department of Intensive Medicine, Facultad de Medicina, Pontificia Universidad Católica de Chile - Santiago, Chile.; 2 HCor-Hospital do Coração Research Institute São Paulo SP Brazil Research Institute, HCor-Hospital do Coração - São Paulo (SP), Brazil.

The use of ventilator-free days (VFDs) as an outcome measure is increasingly popular in critical care research.^(1-3)^ This composite outcome simultaneously reflects patient survival and the time not spent on mechanical ventilation (MV) within a specified timeframe, which usually extends from randomization up to Day 28. For patients who do not survive this period, VFDs are recorded as zero.

Composite outcomes, such as those combining death, myocardial infarction, or stroke, are commonly used in fields such as cardiology due to their ability to enhance the statistical power of clinical trials while focusing on patient-relevant events. In intensive care, where the key outcomes often include the duration of MV, other organ support measures, and hospital stay, the necessity of integrating binary outcomes such as mortality with these continuous variables becomes apparent. The measure of VFDs was proposed more than 20 years ago to effectively merge these outcome types. It has been the most widely used composite outcome in trials of MV and acute respiratory distress syndrome (ARDS). However, in recent years, several authors have called attention about the limitations of VFDs, and potential alternative statistical approaches have been proposed.^(3-5)^

Among the main criticisms of VFDs are that it equals death to remaining on MV for more than 28 days, two outcomes that are valued very differently by patients, relatives, and clinicians.^(3,4)^ Another problem of VFDs is the complexity of calculating a realistic sample size when using it as the primary outcome, as recently shown by Renard Triché et al.^(5)^ Estimating the expected VFDs for a given population can be extremely complex when planning a trial, potentially leading to inadequate decisions about the primary outcome^(6)^ or the determination of an insufficient sample size.^(5)^

An additional challenge is estimating the potential contribution of mortality *versus* duration of MV to the expected differences in VFDs. In 2019, Yehya et al. published a comprehensive discussion about the use of VFDs as an outcome in critical care trials.^(3)^ In that publication, they compared various statistical approaches to analyze VFDs in different potential scenarios of changes in mortality and MV duration. In their study published in Critical Care Science, Serpa-Neto et al. further explored novel statistical approaches to analyze VFDs, such as median regression and cumulative logistic regression. In addition, they analyzed hierarchical composite outcomes such as the win ratio, conditional approaches, or truncated tests, which include mortality and MV duration but prioritize mortality as the most relevant outcome. By running simulations in different potential scenarios involving treatment effects on these outcomes, they analyzed the performance of several statistical approaches to evaluate a composite outcome that integrates mortality and duration of MV.^(7)^ Their analysis offers insight into the complexity of handling the dual aspects of mortality and ventilation duration. This work may have implications not only for trialists but also for clinicians.

For trialists, the findings suggest a nuanced approach to choosing primary outcomes in intensive care trials. Although VFDs present certain limitations, their use may be justified under specific conditions: first, when the mortality rate is too low for mortality alone to serve as a practical primary outcome; second, when there is a reasonable expectation that the intervention will reduce the time spent on MV among survivors combined with a beneficial or neutral effect on mortality. When VFDs or other composite outcomes, which include mortality and duration of MV, are selected as the primary outcome, the simulated scenario results offered by Serpa-Neto et al. can guide the selection of the most appropriate statistical method.^(7)^ Techniques such as Fine and Gray regression, cumulative logistic regression, the win ratio, and the truncated approach demonstrate robust power in scenarios where the treatment effects range from neutral to strong for mortality and from weak to strong for the duration of ventilation. However, if these assumptions are not met, then choosing a composite outcome may lead to a loss in statistical power.

This was the case we recently experienced when planning a multicenter randomized controlled trial comparing prolonged versus intermittent prone positioning for moderate-to-severe ARDS. A prolonged prone position is a strategy aimed at optimizing the beneficial effects of the prone position. During the discussion of the primary outcome selection, we had to choose between aiming at mortality reduction alone or a composite outcome that included the duration of MV. At first glance, a composite approach was appealing for increasing statistical power. However, the PROSEVA trial demonstrated that prone positioning has a strong effect on mortality but no clear effect on the duration of MV^(8)^ (Table 1). Inconsistent treatment effects on mortality and duration of MV have been shown in a number of trials aimed at increasing lung protection in ARDS patients, which have resulted in either beneficial or detrimental impacts on mortality but no change in the duration of MV (e.g., the ARMA, Oscillate, and ART trials)^(9-11)^ (Table 1). In particular, some trials suggest treatment effect on the duration of MV and mortality pointing to opposite directions, such as ARMA (lower mortality but longer duration of MV) and LaSRS (higher mortality but shorter duration of MV).^(9,12)^ If the intervention to be tested decreases mortality but has no effect on the duration of MV, the use of a composite endpoint such as VFDs for the primary outcome, instead of mortality alone, will result in a loss of statistical power.

**Table 1:** Mortality and duration of mechanical ventilation in acute respiratory distress syndrome trials

		ARMA	PROSEVA	ART	OSCILLATE
Intervention		Low Vt	Prone	LRM + PEEP titration	HFOV
Control		High Vt	Supine	Low-PEEP	Conventional ventilation
Mortality on Day 28 (%)					
	Intervention	24	16	55.3	40
	Control	34	32.8	49.3	29
	p value	0.001	< 0.001	0.041	0.004
Duration of MV in survivors (days)*					
	Intervention	8.9 ± 7.1	17 ± 16	16.7 ± 8.5	11 (7 - 19)
	Control	8.6 ± 7.8	19 ± 21	15.7 ± 8.7	10 (6 - 18)
	p value	0.680	0.87	0.24	0.59

ARMA - Respiratory Management in ARDS; PROSEVA - Proning Severe ARDS Patients; ART - Alveolar Recruitment for Acute Respiratory Distress Syndrome Trial; OSCILLATE - Oscillation for Acute Respiratory Distress Syndrome Treated Early trial; Vt - tidal volume; LRM - lung recruitment maneuver; PEEP - positive end-expiratory pressure; HFOV - high-frequency oscillatory ventilation; MV - mechanical ventilation.

* Duration of mechanical ventilation is expressed as the mean ± standard deviation, except for the OSCILLATE trial, for which it is expressed as the median (interquartile range).

For clinicians, the work of Serpa-Neto et al. may facilitate understanding the caveats of interpreting VFDs (and other free-day-related outcomes), as well as their inherent strengths and limitations. First, clinicians should perform a critical appraisal of trials using VFDs and other composite endpoints as the primary outcome to determine which were the previous assumptions that justified this option. Second, instead of just looking at whether the trial was "positive" or "negative", they should routinely scrutinize both the composite outcome and the individual components to gain insights about the real impact of the intervention tested in the trial. This point is particularly relevant for preventing the perception-distortion effect potentially associated with the appraisal of VFDs. This cognitive bias was elegantly introduced by Serpa-Neto et al. to highlight how a relative change in VFDs may lead to under- or overestimation of the clinical relevance of the effects of an intervention if the judgment is based solely on the absolute numbers of VFDs presented, without a thorough examination of the implicit determinants.^(7)^
