# Supplementary material for: Challenges and limitations of using ventilator-free days as an outcome in critical care trials
Source: Crit Care Sci. 2024 Apr 22;36:e20240088en. doi: 10.62675/2965-2774.20240088-en (PMC11098069; doi:10.62675/2965-2774.20240088-en)
Supplement: Translation

# Translation: Desafios e limitações do uso de dias livres de ventilador como desfecho em estudos de cuidados intensivos

**DOI:** 10.62675/2965-2774.20240088-pt

**Authors:** Alejandro Bruhn, Eduardo Kattan, Alexandre Biasi Cavalcanti

**Affiliations:** 1 Santiago Chile Departamento de Medicina Intensiva, Facultad de Medicina, Pontificia Universidad Católica de Chile - Santiago, Chile.; 2 São Paulo SP Brasil Instituto de Pesquisa, HCor-Hospital do Coração - São Paulo (SP), Brasil.

O uso da medida de desfecho dias livres de ventilador (DLVs) é cada vez mais popular em pesquisas sobre cuidados intensivos.^(1-3)^ Esse desfecho composto reflete simultaneamente a sobrevida do paciente e o tempo fora de ventilação mecânica (VM) dentro de um período de tempo específico, que geralmente se estende da randomização até o dia 28. Para os pacientes que não sobrevivem a esse período, os DLVs são registrados como zero.

Os desfechos compostos, como os que combinam morte, infarto do miocárdio ou acidente vascular cerebral, são comumente usados em áreas como a cardiologia, devido à sua capacidade de aumentar o poder estatístico dos estudos clínicos e, ao mesmo tempo, concentrar-se em eventos relevantes para o paciente. Na terapia intensiva, em que os principais desfechos geralmente incluem a duração da VM, outras medidas de suporte de órgãos e a internação hospitalar, torna-se evidente a necessidade de integrar desfechos binários (como mortalidade) a essas variáveis contínuas. Há mais de 20 anos, propôs-se usar DLVs para combinar efetivamente esses tipos de desfechos. Ele tem sido o desfecho composto mais amplamente adotado em estudos sobre VM e síndrome do desconforto respiratório agudo (SDRA). Entretanto, nos últimos anos, vários autores têm se concentrado nas limitações dos DLVs, e possíveis abordagens estatísticas alternativas têm sido propostas.^(3-5)^

Entre as principais críticas aos DLVs está a de considerar a permanência em VM por mais de 28 dias como equivalente à morte, sendo que esses dois desfechos são valorizados de forma muito diferente por pacientes, familiares e clínicos.^(3,4)^ Outro problema dos DLVs é a complexidade de calcular um tamanho de amostra realista ao usá-los como desfecho primário, conforme demonstrado recentemente por Renard Triché et al.^(5)^ Estimar os DLVs esperados para uma determinada população pode ser extremamente complexo no planejamento de um estudo, levando eventualmente a decisões inadequadas sobre o desfecho primário^(6)^ ou à determinação de um tamanho de amostra insuficiente.^(5)^

Outro desafio é estimar a possível contribuição da mortalidade *versus* a duração da VM quanto às diferenças esperadas nos DLVs. Em 2019, Yehya et al. publicaram uma discussão abrangente sobre o uso dos DLVs como um desfecho em estudos de cuidados críticos.^(3)^ Nessa publicação, eles compararam várias abordagens estatísticas para analisar DLVs em diferentes cenários possíveis de mudanças na mortalidade e na duração da VM. Em seu estudo publicado na *Critical Care Science*, Serpa-Neto et al. exploraram ainda mais as novas abordagens estatísticas para analisar os DLVs, como a regressão mediana e a regressão logística cumulativa. Além disso, eles analisaram técnicas para desfechos compostos hierárquicos, como a razão de vitórias (win ratio), abordagens condicionais ou testes truncados, que incluem mortalidade e duração da VM, mas priorizam a mortalidade como o resultado mais relevante. Ao executar simulações em diferentes cenários envolvendo efeitos do tratamento sobre esses desfechos, eles analisaram o desempenho de várias abordagens estatísticas para avaliar um desfecho composto que integra a mortalidade e a duração da VM.^(7)^ Sua análise oferece uma visão da complexidade de lidar com os aspectos duplos da mortalidade e da duração da ventilação. Esse trabalho pode ter implicações não apenas para os pesquisadores, mas também para os clínicos.

Para pesquisadores, os resultados sugerem uma abordagem diferenciada para a escolha de desfechos primários em estudos de terapia intensiva. Embora os DLVs apresentem certas limitações, seu uso pode ser justificado em condições específicas: primeiro, quando a taxa de mortalidade é muito baixa para que a mortalidade por si só sirva como um desfecho primário prático; segundo, quando há uma expectativa razoável de que a intervenção reduzirá o tempo sob VM entre os sobreviventes, combinada com um efeito benéfico ou neutro sobre a mortalidade. Quando os DLVs ou outros desfechos compostos, que incluem mortalidade e duração da VM, são selecionados como desfecho primário, os resultados do cenário simulado oferecidos por Serpa-Neto et al. podem orientar a seleção do método estatístico mais apropriado.^(7)^ Técnicas, como a regressão de Fine e Gray, a regressão logística cumulativa, a razão de vitórias e a abordagem truncada, demonstram um poder robusto em cenários em que os efeitos do tratamento variam de neutro a forte para a mortalidade e de fraco a forte para a duração da ventilação. Entretanto, se essas premissas não forem atendidas, a escolha de um desfecho composto pode levar a uma perda de poder estatístico.

Esse foi o caso que experimentamos recentemente ao planejar um estudo controlado e randomizado multicêntrico comparando o posicionamento prolongado *versus* intermitente em decúbito ventral para SDRA moderada a grave. A posição em decúbito ventral prolongada é uma estratégia que visa otimizar os efeitos benéficos da posição em decúbito ventral. Durante a discussão da seleção do desfecho primário, tivemos que escolher entre buscar apenas a redução da mortalidade ou um desfecho composto que incluísse a duração da VM. A princípio, uma abordagem composta parecia atraente para aumentar o poder estatístico. No entanto, o estudo PROSEVA demonstrou que o posicionamento em decúbito ventral tem um forte efeito sobre a mortalidade, mas nenhum efeito claro sobre a duração da VM^(8)^ (Tabela 1). Efeitos inconsistentes do tratamento quanto à mortalidade e à duração da VM foram demonstrados em vários estudos que visavam aumentar a proteção pulmonar em pacientes com SDRA, o que resultou em impactos benéficos ou prejudiciais sobre a mortalidade, sem alteração na duração da VM (por exemplo, os estudos ARMA, Oscillate e ART)^(9-11)^ (Tabela 1). Mais especificamente, alguns estudos sugeriram que o tratamento afeta a duração da VM e a mortalidade em direções opostas, como o ARMA (menor mortalidade, mas maior duração da VM) e o LaSRS (maior mortalidade, mas menor duração da VM).^(9,12)^ Se a intervenção a ser testada diminuir a mortalidade, mas não tiver efeito sobre a duração da VM, o uso de um desfecho composto, como os DLVs, como desfecho primário, em vez de apenas a mortalidade, resultará em uma perda de poder estatístico.

**Tabela 1:** Mortalidade e duração da ventilação mecânica em ensaios de síndrome do desconforto respiratório agudo

		ARMA	PROSEVA	ART	OSCILLATE
Intervenção		Vt baixo	Decúbito ventral	MRA + titulação de PEEP	VOAF
Controle		Vt alto	Decúbito dorsal	PEEP baixa	Ventilação convencional
Mortalidade no dia 28 (%)					
	Intervenção	24	16	55,3	40
	Controle	34	32,8	49,3	29
	Valor de p	0,001	< 0,001	0,041	0,004
Duração da VM em sobreviventes (dias)*					
	Intervenção	8,9 ± 7,1	17 ± 16	16,7 ± 8,5	11 (7 - 19)
	Controle	8,6 ± 7,8	19 ± 21	15,7 ± 8,7	10 (6 - 18)
	Valor de p	0,680	0,87	0,24	0,59

ARMA - *Respiratory Management in ARDS*; PROSEVA - *Proning Severe ARDS Patients*; ART - *Alveolar Recruitment for Acute Respiratory Distress Syndrome Trial*; OSCILLATE - *Oscillation for Acute Respiratory Distress Syndrome Treated Early trial*; Vt - volume corrente; MRA - manobra de recrutamento alveolar; PEEP - pressão expiratória positiva final; VOAF - ventilação oscilatória de alta frequência; VM - ventilação mecânica.

* A duração da ventilação mecânica é expressa como média ± desvio-padrão, exceto no estudo OSCILLATE, em que é expressa como mediana (intervalo interquartil).

O estudo de Serpa-Neto et al. pode ajudar os clínicos a compreender as peculiaridades da interpretação dos DLVs (e outros desfechos relacionados a tempo não gasto), bem como seus pontos fortes e limitações inerentes. Em primeiro lugar, os clínicos devem efetuar uma avaliação crítica dos ensaios que utilizam DLVs e outros parâmetros compostos, como desfecho primário para determinar quais foram os pressupostos anteriores que justificaram essa opção. Em segundo lugar, em vez de se limitarem a verificar se o ensaio foi "positivo" ou "negativo", devem examinar regularmente tanto o desfecho composto como os componentes individuais, para obterem informações do impacto real da intervenção testada no ensaio. Esse ponto é particularmente relevante para evitar o efeito de distorção da percepção possivelmente associado à avaliação de DLVs. Esse viés cognitivo foi elegantemente introduzido por Serpa-Neto et al. para realçar como uma alteração relativa nos DLVs pode levar a subestimar ou sobrestimar a relevância clínica dos efeitos de uma intervenção, se o julgamento se basear apenas nos números absolutos dos DLVs apresentados, sem exame minucioso dos determinantes implícitos.^(7)^
